# Editorial: Understanding and controlling adverse effects in myeloma patients treated with T cell redirecting immunotherapies

**DOI:** 10.3389/fimmu.2026.1845748

**Published:** 2026-04-23

**Authors:** Jakub Krejcik, Anne Marit Sponaas, Tuna Mutis

**Affiliations:** 1 Department of Haematology, Odense University Hospital, Odense, Denmark; 2 Centre for Cellular Immunotherapy of Haematological Cancer Odense (CITCO), Odense University Hospital, Odense, Denmark; 3 Department of Clinical and Molecular Medicine, Faculty of Medicine and Health Sciences, Norwegian University of Science and Technology (NTNU), Trondheim, Norway; 4 Department of Hematology, Location VUmc, Cancer Center Amsterdam, Amsterdam University Medical Center (UMC), Amsterdam, Netherlands

**Keywords:** bispecific antibodies (BsAbs), CAR T, immunotherapy, infections, multiple myeloma

The notion that the immune system can cure multiple myeloma MM has existed for decades. Early attempts to harness antitumor immunity were associated with limited efficacy, considerable complexity, and substantial toxicity (1). Only in the past decade have strategies to redirect the immune system against malignant plasma cells evolved into therapies that are broadly applicable to most patients and capable of producing highly consistent and durable responses.

Recent phase III studies have demonstrated unprecedented response rates, with high rates of minimal residual disease (MRD) negativity when immunotherapies such as bispecific antibodies or CAR T-cell therapy are used in earlier lines of treatment (2, 3). However, treatment-related toxicities remain a significant challenge (4). Given the remarkable efficacy of these therapies—and their potential to cure a subset of patients—it is critically important to better understand and develop strategies to control toxicity and avoid treatment-related morbidity. This is particularly relevant for cellular therapies, which involve living drugs that cannot simply be paused or discontinued once administered.

Multiple contributions in this Research Topic focus on adverse events associated with immunotherapies in MM and their management. The article by Zhou et al. highlights several emerging safety concerns related to immunotherapy in MM. At the population level, a pharmacovigilance analysis evaluates adverse events associated with bispecific antibody therapies. Notably, the significant safety signals identified are largely consistent with those reported in prior clinical trials. However, each bispecific antibody also demonstrated several novel safety signals, underscoring the need for continued pharmacovigilance and further investigation in real-world settings as these therapies become more widely used in clinical practice.

The next article by Munárriz et al. provides further insight into immune-related toxicities associated with immunotherapy in MM the management of which remains challenging. Among these complications, macrophage activation syndrome–like (MAS-like) toxicity has been increasingly recognized; however, its true incidence remains uncertain due to underreporting and heterogeneous diagnostic criteria. In this context, a multicenter retrospective study conducted by a Spanish group evaluating patients with multiple myeloma treated with an academic anti-BCMA CAR T-cell product offers important insights. In this cohort, 15% of patients met the criteria for MAS-like syndrome, which typically occurred approximately nine days after infusion and was characterized by marked hyperferritinemia, followed by elevations in lactate dehydrogenase (LDH) and hypofibrinogenemia. Importantly, the study also identifies clinical features associated with increased susceptibility to this complication. Patients who developed MAS-like toxicity exhibited more aggressive disease characteristics at baseline and experienced inferior outcomes, including lower complete response rates and shorter progression-free and overall survival. These findings highlight MAS-like toxicity as a clinically relevant and potentially underrecognized complication of CAR T-cell therapy that may also reflect underlying high-risk disease biology. A better understanding of the cellular and molecular mechanism behind this syndrome would also be advantageous.

This issue also includes a detailed case report by Phan et al. describing a previously unreported neurological complication associated with CAR T-cell therapy. The authors present a compelling and carefully documented case of severe cerebral vasculopathy ultimately consistent with reversible cerebral vasoconstriction syndrome (RCVS) in a patient treated with CAR T-cell therapy for relapsed/refractory multiple myeloma. The report provides an important discussion on differentiating RCVS from immune effector cell–associated neurotoxicity syndrome (ICANS) and primary central nervous system vasculitis. It expands the spectrum of recognized neurological toxicities associated with CAR T-cell therapy and highlights the importance of thorough diagnostic evaluation in patients presenting with atypical neurological symptoms.

As novel CAR targets continue to be developed, the risk of on-target, off-tumor toxicities increases, underscoring the need for controllable CAR T-cell systems. In this context, the article by Bielowski et al. explores potential strategies for regulating circulating CAR T cells without affecting endogenous T cells. One of the approaches was to investigate whether antibody dependent cytotoxicity using antibodies targeting engineered antigens on the CAR product could remove CAR T cells. For example, BCMA-targeting antibody–drug conjugate belantamab mafodotin can selectively eliminate BCMA co-expressing CAR T cells while sparing unmodified T cells. This approach suggests that antibody–drug conjugates may represent a promising strategy for controlling CAR T-cell expansion and managing severe toxicities.

Finally, the last two articles in this Research Topic address the important issue of infections during treatment with bispecific antibodies. Traditionally, the increased risk of infection in patients with multiple myeloma has been attributed to impaired function of the residual normal plasma cell population and the associated hypogammaglobulinemia (5). In addition, immunotherapeutic agents used in MM commonly target antigens that are not only expressed by myeloma cells but also by their normal counterparts across different stages of B-cell development (6). As a result, treatment with bispecific antibodies is associated with profound hypogammaglobulinemia and a frequent need for immunoglobulin replacement.

The article by Poulsen et al. focuses specifically on the role of immunoglobulin replacement therapy in patients receiving bispecific antibodies. In this systematic review, the authors identified five retrospective, non-randomized cohort studies comprising a total of 653 patients. Three of these studies reported a significant reduction in infections, particularly severe infections, among patients receiving immunoglobulin replacement therapy. Nevertheless, given the considerable time and resource burden associated with continuous immunoglobulin prophylaxis for both patients and healthcare systems, the authors conclude that further prospective randomized studies are needed to confirm these findings and guide evidence-based practice.

Despite the widespread adoption of immunoglobulin replacement therapy in clinical practice and its potential to reduce the incidence of severe infections, grade 1–2 infections remain a significant issue in many patients. Notably, these infections may persist even after immunoglobulin levels have normalized following the initiation of substitution therapy. One possible explanation is the exhaustion of T-cell immunity, a process that is not easily detectable through routine clinical blood tests. The final article by Dean in this Research Topic addresses the problem of T-cell exhaustion, which may be induced by frequent and continuous dosing of bispecific antibodies. Current dosing schedules are primarily designed to achieve serum drug concentrations known to be effective in *in vivo* tumor-killing assays. However, such approaches may not fully account for the underlying biology of T cells, in which chronic overstimulation can lead to functional exhaustion and reduced effector activity. These findings highlight the importance of optimizing dosing strategies to balance sustained antitumor activity with preservation of immune competence.
